# *Staphylococcal aureus* Enterotoxin C and Enterotoxin-Like L Associated with Post-partum Mastitis

**DOI:** 10.3389/fmicb.2017.00173

**Published:** 2017-02-07

**Authors:** Kristina T. Franck, Heidi Gumpert, Bente Olesen, Anders R. Larsen, Andreas Petersen, Jette Bangsborg, Per Albertsen, Henrik Westh, Mette D. Bartels

**Affiliations:** ^1^Department of Clinical Microbiology, Herlev and Gentofte Hospital, University of CopenhagenHerlev, Denmark; ^2^Department of Clinical Microbiology, Hvidovre Hospital, University of CopenhagenHvidovre, Denmark; ^3^Reference Laboratory for Antimicrobial Resistance and Staphylococci, Statens Serum InstitutCopenhagen, Denmark; ^4^Department of Pediatrics, Nordsjællands Hospital, University of CopenhagenHillerød, Denmark; ^5^Faculty of Health Sciences, Institute of Clinical Medicine, University of CopenhagenCopenhagen, Denmark

**Keywords:** SaPITokyo12571, enterotoxins, clinical presentation, post-partum mastitis, pathogenicity islands, MRSA, whole-genome sequencing, virulence

## Abstract

Denmark is a low prevalence country with regard to methicillin resistant *Staphylococcus aureus* (MRSA). In 2008 and 2014, two neonatal wards in the Copenhagen area experienced outbreaks with a typical community acquired MRSA belonging to the same *spa* type and sequence type (t015:ST45) and both were PVL and ACME negative. In outbreak 1, the isolates harbored SCC*mec* IVa and in outbreak 2 SCC*mec* V. The clinical presentation differed between the two outbreaks, as none of five MRSA positive mothers in outbreak 1 had mastitis vs. five of six MRSA positive mothers in outbreak 2 (*p* < 0.02). To investigate if whole-genome sequencing could identify virulence genes associated with mastitis, t015:ST45 isolates from Denmark (*N* = 101) were whole-genome sequenced. Sequence analysis confirmed two separate outbreaks with no sign of sustained spread into the community. Analysis of the accessory genome between isolates from the two outbreaks revealed a *S. aureus* pathogenicity island containing enterotoxin C and enterotoxin-like L only in isolates from outbreak 2. Enterotoxin C and enterotoxin-like L carrying *S. aureus* are associated with bovine mastitis and our findings indicate that these may also be important virulence factors for human mastitis.

## Introduction

Methicillin resistant *Staphylococcus aureus* (MRSA) is endemic in hospitals worldwide and an increasing challenge in the community. In Denmark, despite a rise in MRSA in the last decade, the prevalence of MRSA is low and is dominated by community-acquired and livestock-associated MRSA (Bager et al., 2015). When MRSA outbreaks occur in Danish hospitals, they are primarily in neonatal wards, where asymptomatic carriage as well as infection is encountered in both neonates and their mothers. The index case, in most outbreaks, is typically a neonate with a minor infection such as conjunctivitis, with the MRSA most likely acquired from a parent. In the Capital Region of Denmark, all MRSA isolates are whole-genome sequenced and *spa*-types, multilocus sequence types (MLST) plus the presence or absence of the Panton-Valentine leukocidin (PVL) genes are reported to the clinicians (Bartels et al., 2015). Apart from the PVL genes, isolates are not routinely examined for other virulence genes such as enterotoxins and exfoliative toxins (Peacock et al., 2002).

We analyzed the genomes of two seemingly similar neonatal MRSA outbreaks caused by MRSA t015:ST45. The clinical presentation of the two outbreaks was, however, very different. Mothers in the first outbreak (O1) were only colonized with MRSA whereas most mothers in the second outbreak (O2) suffered from mastitis caused by MRSA. Outbreaks of post-partum mastitis or post-partum breast abscesses are rarely reported (Saiman et al., 2003; Manoharan et al., 2012) although cohort studies show that 8–20% of breastfeeding women may suffer from mastitis (Scott et al., 2008; Cullinane et al., 2015; Khanal et al., 2015).

## Materials and methods

### Setting

Both outbreaks occurred in the greater Copenhagen area, the Capital Region of Denmark, in 2008 and 2014, respectively, in two different hospital neonatal wards. Isolates were found in clinical samples (one case of conjunctivitis, five breast milk samples from patients with mastitis) or through screening for colonization. O1 involved 9 neonates, 5 mothers, and 4 fathers while O2 involved 12 neonates, 6 mothers, and 2 hospital staff members. Fathers were not tested for MRSA in O2. In both outbreaks, MRSA positive neonates and parents were contact isolated. Both wards were disinfected with manual bleach cleaning followed by a hydrogen-peroxide disinfectant mist. MRSA colonized staff members went on medical leave and underwent successful decolonization therapy. The neonates and their parents were not offered decolonization therapy in accordance with the Danish National Guidelines, recommending no treatment until the child turns 2 years (Vejledning om forebyggelse af spredning af MRSA, 2016).

### Data set

MRSA isolates from the outbreaks were investigated by whole-genome sequencing (WGS) as part of the routine at the Department of Clinical Microbiology, Hvidovre Hospital. In order to investigate the relatedness to other Danish MRSA with *spa*-type t015 isolated in 2008-15, WGS from isolates obtained through the national surveillance at Statens Serum Institut, were included in the analysis. The combined data set included 102 patient unique t015:ST45 isolates from all over Denmark isolated in the period 2008–2015. One isolate was excluded due to poor sequencing quality.

### Whole-genome sequencing and analysis

DNA was extracted from isolates and sequencing libraries were prepared using Illumina Nextera XT protocol, according to the manufacturer (Illumina, CA, USA). Isolates were assembled using Velvet (Zerbino and Birney, 2008) with VelvetOptimizer. Isolates were annotated using PROKKA (Seemann, 2014), and the core- and pan-genomes were determined using default values. The aligned, ungapped core genome was used for SNP calling and phylogenetic analysis was performed by RAxML with 100 bootstrap support replicates (Stamatakis, 2014). The resulting phylogenetic tree and accessory genome (non-unique genes present in <70% of isolates) was visualized in R using ggplots2 and ggtree packages (Yu et al., 2017). The columns in the heatmap were ordered by a hierarchical clustering algorithm based on gene presence/absence in the accessory genome using the Jaccard distance metric.

In addition to the pan-genome analysis, regions unique to either of the outbreaks were identified as follows. Reads from the index isolate from O1 were aligned to the genome assembly of the index isolate from O2 using Bowtie2 (Langmead and Salzberg, 2012), and vice versa. Coverage breadth of the read alignment was established using BedTools (Quinlan and Hall, 2010) for coding regions identified from a RAST annotation (Aziz et al., 2008). The coverage breadth output was filtered such that at least 90% coverage breadth of the coding region was required for the gene to be considered present. In this way, consecutive regions of coverage breadth <90% were identified and represented unique genomic regions to one of the index outbreak isolates. The same analysis was performed for the isolates within each outbreak to the index isolate of the outbreak to identify any other difference over the course of the respective outbreaks.

The *spa*, MLST, PVL genes, and SCC*mec* type were extracted from WGS data, as previously described (Bartels et al., 2015). Virulence and resistance genes and plasmid replicons were identified with the online tools VirulenceFinder (Joensen et al., 2014), ResFinder (Zankari et al., 2012), and PlasmidFinder (Carattoli et al., 2014), respectively, available from the homepage http://genomicepidemiology.org/.

The sequences of the index isolates from each outbreak have been deposited at NCBI under WGS accession numbers MPPG00000000 and MPPH00000000.

### Statistical analysis

Fisher's exact test was used for comparison of the proportions of mastitis among mothers in the two outbreaks.

### Ethical considerations

The study was approved by the Danish Data Protection Agency (no. 2012-58-0004).

## Results

### MRSA colonization and infection

In O1, the index case was a neonate who tested MRSA positive in a conjunctivitis sample. The remaining 17 MRSA positive cases were colonized without infection and there were no reports of mastitis in the five MRSA positive mothers. In O2, the index case was a mother with mastitis. MRSA as well as MSSA was detected in a sample of her breast milk. Twelve neonates, six mothers and two staff members tested MRSA positive. A significant larger proportion of the MRSA positive mothers in O2 suffered from early onset mastitis based on results from culturing of breast milk samples (5/6) compared to MRSA positive mothers in O1 (0/5) (*p* < 0.02).

### Whole-genome sequencing results

**Figure 1:**
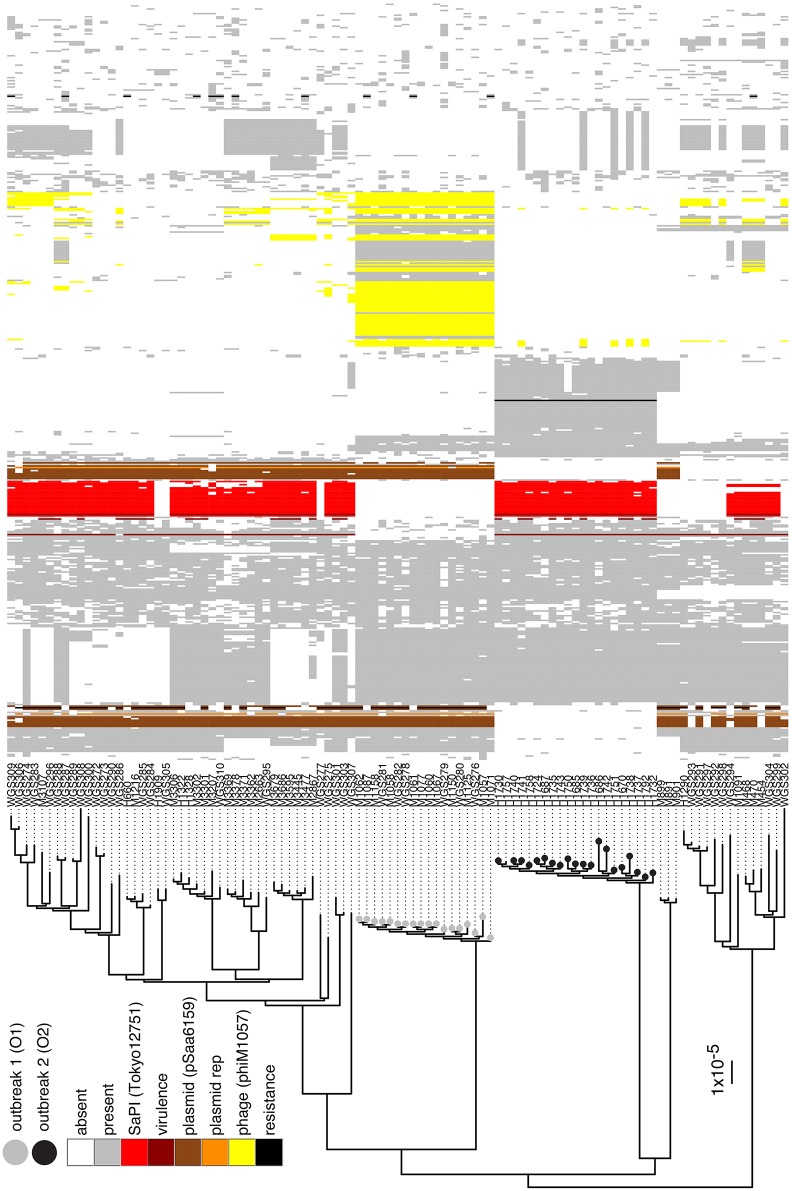
**Phylogenetic tree and accessory genome heatmap**. On the left hand side is a maximum-likelihood-based phylogenetic tree based on the variable positions in the core-genome of the t015:ST45 isolates from Denmark during 2008–2015. Scale bar indicates the substitution rate. Isolates with a gray circle belong to O1 and black circle to O2. On the right is a heat map of the accessory genome of O1 and O2 isolates, where the accessory genome is defined as the genes that are present in at most 70% of the isolates. Columns are sorted by a hierarchal clustering algorithm based on gene absence/presence in the O1 and O2 accessory genome. Genes that have been identified to belong to the SaPI present in O2, the multi-replicon plasmid in O1, phages in O1 and O2 are colored, along with virulence and resistance genes and plasmid replicons identified by VirulenceFinder, ResFinder, and PlasmidFinder, respectively.

High quality WGS data were obtained for 101 (99%) of the MRSA isolates. All isolates were confirmed to be *spa* type t015:ST45. No isolates were PVL-positive. A phylogenetic tree based on the ungapped alignment of core-genome (2095 genes totaling 1,655,427 bp) of the isolates was produced to investigate the relationship between the isolates (Figure 1). The average number of base pair substitutions in the core genome of all the isolates was 340. Isolates from O1 and O2 grouped into two distinct, separate clusters with isolates within O1 and O2 differing by no more than 22 and 44 positions, respectively. There were a minimum of 420 variable positions in the core genome between isolates from O1 and O2. The O1 cluster contained only isolates from known outbreak patients, whereas an additional isolate sampled from a woman with no known contact to the outbreak grouped into the O2 cluster (H1757). This patient was a 53 year old woman who lived in the catchment area of the hospital, where O2 occurred.

We further investigated whether differences in the genomic content between isolates of the two outbreaks might contribute to the enhanced virulence (based on the number of mastitis cases) observed in O2 compared to O1. Isolates from O2 were found to harbor SaPITokyo12571 (Suzuki et al., 2015), a pathogenicity island that contained genes encoding staphylococcal enterotoxin C (SEC) and staphylococcal enterotoxin-like L (SElL; Figure 1). VirulenceFinder identified the *sec* and *sell* genes as the only difference in known virulence factors between the two outbreak index isolates (Supplementary Table). Further analysis of 4695 *S. aureus* genomes in our database revealed that 562 isolates (12%) harbored both *sec* and *sell* genes while only 10 isolates (0.2%) had *sell* alone. Among the 562 isolates, 120 belonged to ST45 and 71% of all ST45 isolates in the complete WGS database harbored *sec* and *sell*. None of the ST45 isolates harbored the PVL genes. Isolates from O1 harbored a rep5-rep16 multi-replicon 20.8 kbp plasmid that shared 99% identity with pSaa6159 (Chua et al., 2010). This plasmid harbored cadmium resistance genes, and *blaZ* beta-lactamase gene with corresponding *blaR1* and *blaI* regulatory genes. Additionally, O1 isolates harbored an extra 42.8 kbp on the chromosome relative to O2 isolates due to insertion of a 33.8 kbp prophage and partly from the different sizes of the two SCC*mec* cassettes (SCC*mec* IVa in O1 and SCC*mec* V in O2). This prophage, phiM1057, most closely resembles bacteriophage 29, (GenBank: AY954964) with a coverage of about 80% and also has the same integrase.

The distribution of the accessory genome of the O1 and O2 isolates across all t015 isolates in the study revealed that while some elements were unique to one of the outbreaks (e.g., phiM1057 in O1), others were relatively widespread across the other t015 isolates (e.g., the multireplicon plasmid from O1 and the SaPITokyo12571 from O2; Figure 1).

## Discussion

Human outbreaks of post-partum mastitis and transmission between mothers with MRSA in breast milk and neonates have been described previously (Kawada et al., 2003; Saiman et al., 2003; Behari et al., 2004; Sanchini et al., 2013). In these outbreaks, the infecting *S. aureus* strains expressed the PVL genes, a known cause of skin and soft tissue infections. None of our outbreak strains contained the PVL genes. The two outbreaks, although having identical *spa*-type and ST (t015:ST45), were found to be caused by different MRSA and a possible cause of mastitis in O2 was identified as a SaPI similar to SaPITokyo12571. This SaPI contained *sec* and *sell*, which have both been described as individual causes of mastitis in cows (Kenny et al., 1993; Orwin et al., 2003; Artursson et al., 2016). SEC has also been detected in a PVL containing MRSA strain causing an outbreak of skin-and soft tissue infections including mastitis among post-partum women (Saiman et al., 2003). *sec* and *sell* were the only additional known virulence genes identified by VirulenceFinder in O2 compared to O1. Our finding that *sec* and *sell* are in the genome of 12% of our MRSA isolates, with *sell* alone in 0.2%, suggests a connection between either or both enterotoxins with the development of mastitis in women. Interestingly, *sec* and *sell* were found in 71% of the ST45 isolates in our WGS database and none of the ST45 isolates harbored the PVL genes. *sec* and *sell* were found to be seven times more common in ST45 than in the remaining MRSA clonal complexes. The prophage, phiM1057, identified in O1, was in contrast only associated to this outbreak and not seen in other ST45 isolates. The plasmid, pSaa6159, was common in ST45 isolates but not found in O2 and contained no known virulence genes.

With this study, we used WGS on nationally collected MRSA strains to document the containment of two outbreaks of MRSA in two neonatal wards. In one outbreak, the majority of mothers developed mastitis, which could be associated with the presence of a *S. aureus* pathogenicity island containing *sec* and *sell* in the outbreak strain. Further analysis of WGS data in future outbreaks can pinpoint genes that can cause specific clinical presentations.

## Author contributions

KF, BO, AL, AP, HW, MB: conceived the study. KF, HG, AL, AP, HW, MB: analyzed and interpreted the results. KF, BO, AL, AP, JB, PA, HW, MB: collected and interpreted data. KF, HW: drafted the manuscript, which was critically revised and final approved by all the authors.

### Conflict of interest statement

The authors declare that the research was conducted in the absence of any commercial or financial relationships that could be construed as a potential conflict of interest.
