# Supplementary material for: Staphylococcal aureus Enterotoxin C and Enterotoxin-Like L Associated with Post-partum Mastitis
Source: Front Microbiol. 2017 Feb 7;8:173. doi: 10.3389/fmicb.2017.00173 (PMC5293744; doi:10.3389/fmicb.2017.00173)
Supplement: Supplementary file 1 [file Table1.DOCX]

Supplementary Table

*Staphylococcal aureus* enterotoxin C and enterotoxin-like L associated with post-partum mastitis

**Kristina Træholt Franck^*^, Heidi Gumpert, Bente Olesen, Anders Rhod Larsen, Andreas Petersen, Jette Bangsborg, Per Albertsen, Henrik Westh, Mette Damkjær Bartels**

*** Correspondence:** Corresponding Author: [frk.franck@dadlnet.dk](mailto:frk.franck@dadlnet.dk)

List of genes identified by VirulenceFinder in the index isolates from outbreak 1 and outbreak 2

| **Outbreak 1 (M1057)** | |  | **Outbreak 2 (H1670)** | |
| --- | --- | --- | --- | --- |
| Genes | Products |  | Genes | Products |
| aur | aureolysin |  | aur | Aureolysin |
| hlb | beta-hemolysin |  | hlb | beta-hemolysin |
| hlgA | gamma-hemolysin chain II precursor |  | hlgA | gamma-hemolysin chain II precursor |
| hlgB | gamma-hemolysin component B precursor |  | hlgB | gamma-hemolysin component B precursor |
| hlgC | gamma-hemolysin component C |  | hlgC | gamma-hemolysin component C |
| sak | staphylokinase |  | sak | staphylokinase |
| scn | staphylococcal complement inhibitor |  | scn | staphylococcal complement inhibitor |
| seg | enterotoxin G |  | **sec3** | **enterotoxin C** |
| sei | enterotoxin I |  | seg | enterotoxin G |
| sem | enterotoxin M |  | sei | enterotoxin I |
| sen | enterotoxin N |  | **sel** | **enterotoxin L** |
| seo | enterotoxin O |  | sem | enterotoxin M |
| seu | enterotoxin U |  | sen | enterotoxin N |
|  |  |  | seo | enterotoxin O |
|  |  |  | seu* | enterotoxin U |

*The assembly contained the gene on two contig ends
